# Supplementation of Yeast Cell Wall Fraction Tends to Improve Intestinal Health in Adult Dogs Undergoing an Abrupt Diet Transition

**DOI:** 10.3389/fvets.2020.597939

**Published:** 2020-11-11

**Authors:** Ching-Yen Lin, Meredith Q. Carroll, Michael. J. Miller, Rodolphe Rabot, Kelly S. Swanson

**Affiliations:** ^1^Division of Nutritional Sciences, University of Illinois at Urbana-Champaign, Urbana, IL, United States; ^2^Department of Animal Sciences, University of Illinois at Urbana-Champaign, Urbana, IL, United States; ^3^Department of Food Science and Human Nutrition, University of Illinois at Urbana-Champaign, Urbana, IL, United States; ^4^Phileo by Lesaffre, Marcq-en-Barœul, France

**Keywords:** canine nutrition, fecal characteristics, gut stability, intestinal immunity, stool quality

## Abstract

When owners decide to change their pet's food, a rapid transition may cause gastrointestinal distress. Yeast products may help with digestive upset during diet transition due to the bioactive compounds they possess, which may lead to improved intestinal morphology and integrity, modified gut microbiota, and modulated immune responses. The objective of this study was to determine the effects of a yeast cell wall fraction supplement on measures of gut integrity and fecal characteristics of adult dogs undergoing an abrupt diet transition. Twelve adult female beagles (mean age: 5.16 ± 0.87 years; mean body weight: 13.37 ± 0.68 kg) were used in a replicated 4 × 4 Latin square design with four 28-day experimental periods. During days 1–14, dogs were fed a dry kibble diet and supplemented with a placebo (cellulose; 125 mg/d) or yeast product (365 mg/d; equivalent to 0.2% of diet). During days 15–28, dogs remained on their placebo or yeast treatments but were rapidly transitioned to a canned diet or high-fiber diet. Fresh fecal samples were collected on days 13, 16, 20, 24, and 27 for measurement of pH, dry matter, calprotectin, immunoglobulin A (IgA), *Escherichia coli*, and *Clostridium perfringens*. Blood samples were collected on days 14, 17, and 28 to measure serum lipopolysaccharide-binding protein concentrations. All data were analyzed using the Mixed Models procedure of SAS 9.4. Fecal pH, dry matter, calprotectin, IgA, and *E. coli* were not affected (*P* > 0.05) by treatment before diet transition. Dogs supplemented with yeast cell wall fraction tended to have higher (*P* = 0.06) fecal *C. perfringens* counts than the controls. After diet transition, most parameters were not altered (*P* > 0.05) by treatment except that yeast-supplemented dogs tended to have higher (*P* = 0.06) fecal IgA than controls. Our results suggest that the yeast product may modestly improve intestinal health after an abrupt diet transition in adult dogs by enhancing intestinal immunity.

## Introduction

Diet transition commonly occurs in dogs when owners purchase new products. If not transitioned slowly, digestive upset may occur, which results in poor stool quality and gastrointestinal (GI) upset. Based on past research, the supplementation of functional ingredients such as yeast products may serve as a potential approach to minimizing digestive upset in such situations.

Yeast products are ingredients containing yeast cells or yeast derivatives and are commonly used in livestock feeds to improve animal performance (1, 2). Yeast products have also been shown to improve GI health in various ways. For example, dogs (3–6) and cats (7) supplemented with yeast products had reduced potential pathogenic bacteria (e.g., *Escherichia coli*; *Clostridium perfringens*) and increased beneficial bacteria (e.g., *Lactobacillus*; *Bifidobacterium*) in feces. In pigs (2, 8) and chickens (1), supplementation of yeast products has led to increased small intestinal villus height and/or villus height: crypt depth ratio. Yeast supplementation enhanced colonic barrier function and decreased intestinal permeability in murine intestinal obstruction and colitis models (9, 10). Lastly, yeast-supplemented dogs (4, 11) and pigs (12) had greater small intestinal immunoglobulin A (IgA) concentrations vs. controls.

Results from those studies indicate that supplementation of yeast products may improve GI health by modifying gut microbiota, improving intestinal morphology and integrity, and/or modulating immune responses. In this study, we aimed to determine the effects of a yeast cell wall fraction product on measures of GI tract stability and fecal characteristics of adult dogs undergoing an abrupt diet transition. We hypothesized that yeast cell wall fraction-supplemented dogs would have greater gastrointestinal stability and protection from a disrupted gut barrier function due to abrupt diet change, as well as reduced fecal pathogens compared to control dogs receiving a placebo treatment.

## Materials and Methods

### Animals, Diets, and Treatments

All animal care and experimental procedures were approved by the University of Illinois Institutional Animal Care and Use Committee (Protocol No. 17276) prior to experimentation. All methods were performed in accordance with the United States Public Health Service Policy on Humane Care and Use of Laboratory Animals. Twelve adult female beagles (mean age: 5.16 ± 0.87 years; mean BW: 13.37 ± 0.68 kg) were used and housed individually in pens (1.0 m wide × 1.8 m long) in a humidity- and temperature-controlled room on a 14 h light: 10 h dark cycle. Dogs had access to fresh water *ad libitum* at all times. All diets were offered to dogs once a day in the morning to maintain body weight throughout the study. Dogs were weighed and body condition scores were assessed using a 9-point scale (13) weekly before feeding.

A replicated 4 × 4 Latin square design was conducted. Each 28-d experimental period consisted of an adaptation phase (d 1–14) and a diet transition phase (d 15–28). Three diets that met all Association of American Feed Control Officials (AAFCO) (14) nutrient recommendations for adult dogs at maintenance were fed, which included (1) a baseline diet (a dry kibble experimental diet; Tables 1, 2); (2) a commercial canned diet (CD; Ol' Roy Cuts in Gravy with Savory Beef; Walmart, Bentonville, AR; Table 2); and (3) a high-fiber diet (HFD) composed of the experimental diet plus 22.5 g/d of soluble corn fiber (Nutriose® Soluble Digestion-Resistant Prebiotic Corn Fiber; Roquette America Inc., Geneva, IL) that was top-dressed on the diet just prior to feeding. Treatments were given to dogs via gelatin capsules (d 1–28) prior to the diet each day, which included a placebo (125 mg cellulose/d) or yeast cell wall fraction (Safmannan® Phileo by Lesaffre, Marcq-en-Barœul, France; 365 mg/d; equivalent to 0.2% of diet). The yeast cell wall fraction tested was from *Saccharomyces cerevisiae* and the analyzed chemical composition is presented in Table 3. During the adaptation phase, all dogs were fed the experimental diet and supplemented with placebo or yeast product. During the diet transition phase, dogs remained on their placebo or yeast treatments, but were fed their new diet (CD or HFD). Therefore, dogs were allotted to one of four groups each experimental period: (1) Yeast CD; (2) Yeast HFD; (3) Placebo CD; and (4) Placebo HFD.

**Table 1:** Ingredient composition of the experimental diet fed to yeast- or cellulose-supplemented dogs.

**Ingredient**	**Amount (%, as-is)**
Brewer's rice	45.26
Chicken by-product meal	32.00
Chicken fat	9.00
Corn	6.00
Cellulose	6.00
Salt	0.50
Potassium chloride	0.45
Taurine	0.30
Mineral premix^a^	0.18
Vitamin premix^b^	0.18
Choline chloride	0.13

a *Provided per kg diet: Mn (as MnSO_4_), 66.0 mg; Fe (as FeSO_4_), 120.0 mg; Cu (as CuSO_4_), 18.0 mg; Co (as CuSO_4_), 1.20 mg; Zn (as ZnSO_4_), 240.0 mg; I (as KI), 1.80 mg; Se (as Na_2_SeO_3_), 0.24 mg*.

b *Provided per kg diet: vitamin A, 5.28 mg; vitamin D_3_, 0.04 mg; vitamin E, 120.0 mg; vitamin K, 0.88 mg; thiamine, 4.40 mg; riboflavin, 5.72 mg; pantothenic acid, 22.0 mg; niacin, 39.6 mg; pyridoxine, 3.52 mg; biotin, 0.13 mg; folic acid, 0.44 mg; vitamin B_12_, 0.11 mg*.

**Table 2:** Analyzed chemical composition of the experimental diet and canned diet (CD) fed to yeast- or cellulose-supplemented dogs.

**Item**	**Experimental diet**	**Canned diet**
Dry matter (DM; %)	92.82	23.90
	%, of DM	
Crude protein	28.30	41.27
Acid-hydrolyzed fat	14.36	24.35
Total dietary fiber	15.98	11.72
Ash	5.95	12.96
Nitrogen-free extract^a^	35.41	9.72
Metabolizable energy^b^ (kcal/g)	3.51	3.91

a *Nitrogen-free extract (%) = 100% – (crude protein% + acid-hydrolyzed fat% + total dietary fiber% + ash%)*.

b *Metabolizable energy = 3.5 kcal/g × crude protein (%) + 8.5 kcal/g × acid-hydrolyzed fat (%) + 3.5 kcal/g × nitrogen-free extract (%)*.

**Table 3:** Analyzed chemical composition of the yeast cell wall fraction tested.

**Item**	**%**	**Free sugars (mg/g)**	**Hydrolyzed monosaccharides corrected for free sugars (mg/g)**
Dry matter (DM; %)	95.36		
	%, of DM		
Ash	4.78		
Crude protein	14.65		
Acid-hydrolyzed fat	9.28		
Total dietary fiber	57.47		
Soluble fiber	53.38		
Insoluble fiber	4.10		
Arabinose		0.00	0.00
Fructose		0.00	0.00
Galactose		0.00	0.00
Glucose		0.13	227.35
Inositol		32.78	0.00
Mannose		2.44	242.46
Sorbitol		0.15	11.60
Xylose		0.00	0.00

### Fecal Sample Collection

On d 13, 16, 20, 24, and 27 of each experimental period, one fresh fecal sample from each dog was collected within 15 min of defecation. Fecal samples were scored according to a 5-point scale: 1 = hard, dry pellets, small hard mass; 2 = hard, formed, dry stool; remains firm and soft; 3 = soft, formed, and moist stool, retains shape; 4 = soft, unformed stool, assumes shape of container; and 5 = watery, liquid that can be poured. Fecal pH was measured immediately using an AP10 pH meter (Denver Instrument, Bohemia, NY) equipped with a Beckman Electrode (Beckman Instruments Inc., Fullerton, CA). An aliquot of fresh feces was dried at 105°C for 2 d for dry matter (DM) determination.

On d 13, 20, and 27 of each experimental period, two more aliquots of fresh fecal samples were collected for determination of fecal IgA, calprotectin and potential pathogenic bacteria (*E. coli* and *C. perfringens*). For fecal IgA and calprotectin measurements, the aliquot was stored at −80°C until analyses. For fecal bacteria analyses, the aliquot was processed for bacterial culture immediately after collection.

### Fecal IgA and Calprotectin

Fecal proteins were extracted according to Vilson et al. (15). Fecal samples (250 mg) were vortexed with 750 μl extraction buffer containing 50 mM-EDTA (ThermoFisher, Waltham, MA, United States) and 100 μg/l soybean trypsin inhibitor (Sigma, St. Louis, MO, United States) in phosphate-buffered saline/1 per cent bovine serum albumin (Tocris Bioscience, Bristol, UK). Phenylmethanesulphonyl fluoride (12.5 μl, 350 mg/l; Sigma, St. Louis, MO) was added to each tube, followed by centrifugation at 10,000 × g at 4°C for 10 min. The supernatants were collected for measurements of IgA and calprotectin using canine-specific commercial ELISA kits (IgA: # E-40A, Immunology Consultants Laboratory, Portland, OR; calprotectin: #MBS030023, MyBioSource, San Diego, CA, United States).

### Fecal *E. coli* and *C. perfringens*

Fecal *E. coli* and *C. perfringens* were measured using standard culture methods. Briefly, 50 mg feces were homogenized in 1 mL PBS followed by 10^−1^ to 10^−7^ serial dilutions. Diluted fecal samples were inoculated onto petri dishes with bacterial selective agar. The selective agar media used in this study were M-TEC agar (HiMedia Laboratories, Kelton, PA, United States) for *E. coli* and Perfringens OPSP agar (ThermoFisher, Waltham, MA, United States) for *C. perfringens*. *E. coli* was incubated aerobically at 37°C for 12–48 h, while *C. perfringens* was incubated anaerobically at 37°C for 12–48 h. Colony forming units were enumerated after incubation.

### Blood Sample Collection and Serum Lipopolysaccharide (LPS)-Binding Protein

On d 14, 17, and 28 of each experimental period, a fasted blood sample was collected via jugular puncture and transferred to serum tubes containing a clot activator and gel for serum separation (no. 367986, Becton Dickinson, Franklin Lakes, NJ, United States). Serum was separated and collected by centrifuging blood tubes at 1,300 × g at 4°C for 10 min (Beckman CS-6R centrifuge; Beckman Coulter Inc., Brea, CA). Serum lipopolysaccharide-binding protein was measured using a canine-specific commercial ELISA kit (#MBS093112, MyBioSource, San Diego, CA, United States).

### Chemical Analysis of Diets and Yeast Cell Wall Fraction

The CD was dried at 55°C in a forced-air oven for approximately 1 wk. Both diets were then subsampled and ground through a Wiley mill (model 4, Thomas Scientific, Swedesboro, NJ, United States) through a 2 mm screen. Diets and yeast cell wall fractions were analyzed for DM, organic matter (OM), crude protein, acid-hydrolyzed fat, and total dietary fiber (TDF). Dry matter and OM were analyzed according to Association of Official Analytical Chemists (AOAC, 2006; DM: methods 934.01; OM: 942.05) (16). Crude protein content was calculated from Leco (FP2000 and TruMac) total nitrogen values according to AOAC (2006, method 992.15) (16). Fat content was determined using acid hydrolysis methods of the American Association of Cereal Chemists (17) and lipid extraction by Budde (18). Total dietary fiber content was determined according to Prosky et al. (19). Nitrogen free extract (NFE) was calculated using the following equation: 100% – (crude protein% + acid-hydrolyzed fat% + TDF% + ash%). Monosaccharide composition of the yeast cell wall fraction was analyzed according to Bourquin et al. (20).

### Statistical Analysis

For the baseline samples, data were analyzed using the Mixed Models procedure of SAS (version 9.4; SAS Institute, Cary, NC, United States) to test the main effect of treatment, with treatment as a fixed effect and dog as a random effect. For post-diet transition samples, data were expressed as change from baseline and analyzed using the Mixed Models procedure of SAS (version 9.4; SAS Institute, Cary, NC, United States) as repeated measures. The main effects of treatment, diet and time as well as interactions of diet and treatment, diet and time, and treatment and time were tested, with dog as a random effect. Data are reported as means ± standard error of the mean (SEM) with statistical significance set as *P* ≤ 0.05 and trends set as 0.05 < *P* ≤ 0.10.

## Results

### Food Intake and Body Weight

**Figure 1:**
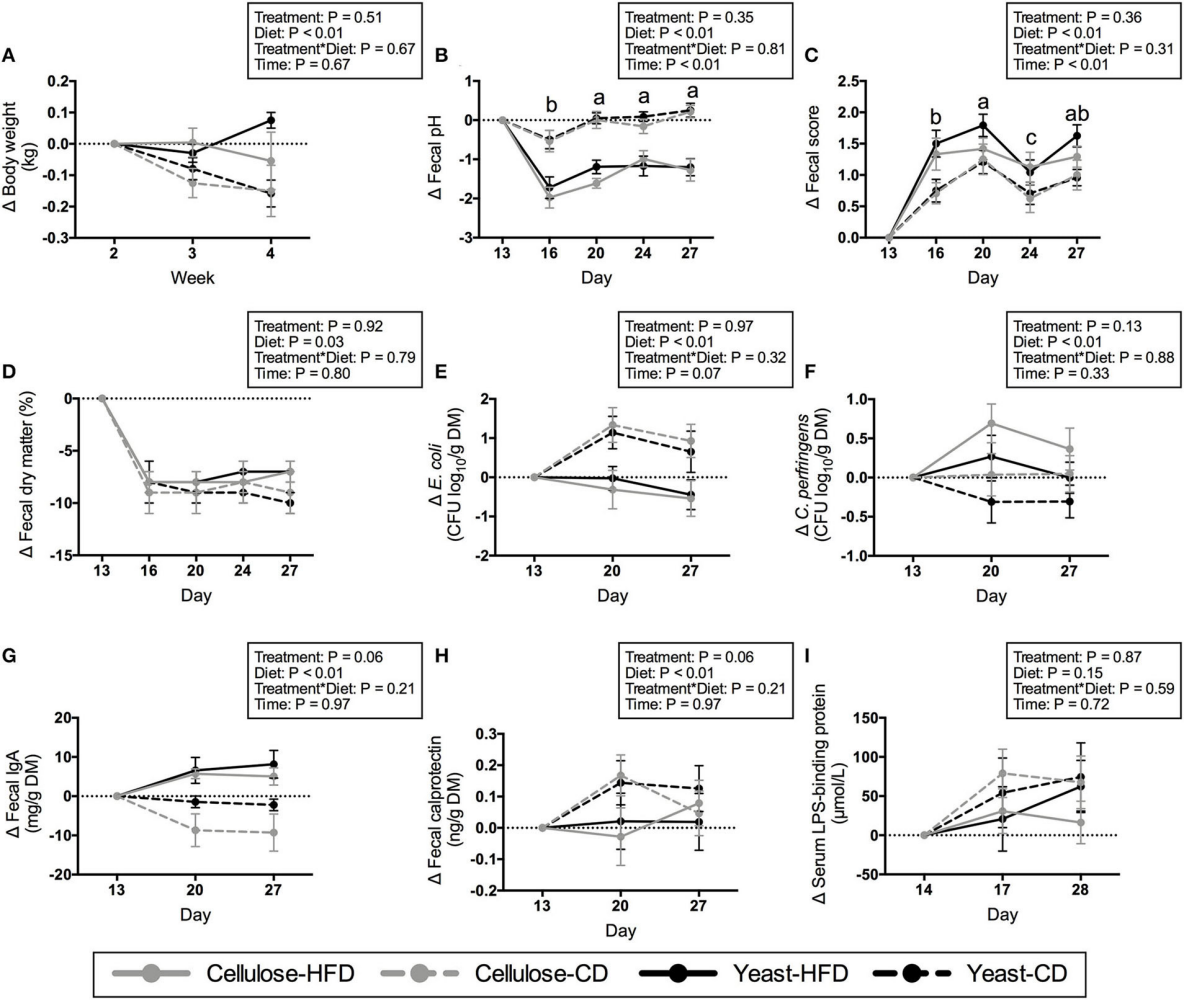
Changes in body weight **(A)**, fecal pH **(B)**, fecal score **(C)**, fecal dry matter **(D)**, fecal *E. coli* **(E)** and *C. perfringens* **(F)** populations, fecal IgA concentration **(G)**, fecal calprotectin concentration **(H)**, and serum lipopolysaccharide-binding protein concentration **(I)** of yeast- or cellulose-supplemented dogs transitioned to a high-fiber diet (HFD) or canned diet (CD). Values represent mean ± SEM changes from baseline (day 13 or 14). ^a,b,c^Mean values within a day with unlike letters were different (*P* < 0.05).

Average daily food, energy, and nutrient intakes, including protein, fat, TDF, and NFE were not changed by yeast treatment prior to diet transition (Table 4). Post-transition diets affected nutrient intakes. Daily intakes of protein, fat, and energy were higher (*P* < 0.01), while daily intakes of TDF and NFE were lower (*P* < 0.01) in dogs fed the CD than those fed the HFD (Table 5). Average BW (13.52 kg) and body condition score (7.15/9) were not altered by yeast treatment prior to and after diet transition (Table 4; Figure 1A). Body weight was affected by diet after diet transition. Dogs consuming CD had greater decreases (*P* < 0.01) in BW than dogs consuming HFD (Figure 1A).

**Table 4:** Daily food, nutrient, and energy intakes, body weight, and body condition score of yeast- or cellulose-supplemented dogs before diet transition^a^.

	**Treatment**			
**Item**	**Cellulose**	**Yeast**	**Pooled SEM**	***P*-value**
Daily intake (g/d)				
Food, as-is	177.3	180.2	3.98	0.22
Food, DMB^b^	164.6	167.3	3.69	0.22
Protein, DMB	46.6	47.3	1.04	0.23
Fat, DMB	25.5	25.9	0.57	0.22
Total dietary fiber, DMB	26.3	26.7	0.59	0.22
Nitrogen-free extract, DMB	56.5	57.4	1.27	0.22
Energy (kcal/d)	577.1	586.5	12.95	0.22
Body weight (kg)	13.51	13.53	0.06	0.78
Body condition score^c^	7.17	7.13	0.06	0.38

a *Data are expressed as means ± pooled standard error of the mean, n = 24*.

b *DMB, dry matter basis*.

c *A 9-point scale body condition scoring system was used (13)*.

**Table 5:** Daily food, nutrient, and energy intakes of yeast- or cellulose-supplemented dogs transitioned to a high-fiber diet (HFD) or canned diet (CD)^a^.

**Item**	**Cellulose**		**Yeast (375 mg/d)**		***P*****-value**		
	**HFD**	**CD**	**HFD**	**CD**	**Treatment**	**Diet**	Treatment *Diet
Daily intake (g/d)							
Food, as-is	177.0 ± 4.62	695.2 ± 12.91	175.9 ± 4.44	678.7 ± 27.08	0.48	<0.01	0.53
Food, DMB^b^	164.2 ± 4.28	160.8 ± 2.99	163.3 ± 4.13	157.0 ± 6.26	0.43	0.11	0.64
Protein, DMB	46.5 ± 1.21	66.4 ± 1.23	46.2 ± 1.17	64.8 ± 2.58	0.43	<0.01	0.57
Fat, DMB	25.4 ± 0.66	40.8 ± 0.76	25.3 ± 0.64	39.9 ± 1.59	0.43	<0.01	0.56
Total dietary fiber, DMB	44.2 ± 0.68	18.8 ± 0.35	44.1 ± 0.66	18.4 ± 0.73	0.47	<0.01	0.73
Nitrogen-free extract, DMB	56.4 ± 1.47	13.9 ± 0.26	56.0 ± 1.41	13.6 ± 0.54	0.68	<0.01	1.00
Energy (kcal/d)	575.8 ± 15.02	628.1 ± 11.66	572.4 ± 14.46	613.1 ± 24.47	0.43	<0.01	0.62

a *Data are expressed as means ± standard error of the mean, n = 12 for HFD and CD*.

b *DMB, dry matter basis*.

### Fecal Characteristics

Fecal characteristics, including pH, score, and DM were not altered by yeast treatment prior to diet transition (Table 6). After diet transition, the change from baseline values for fecal characteristics were affected by diet and time, but not yeast treatment (Figure 1). Fecal pH decreased after diet transition. The change from baseline values for fecal pH were greater (*P* < 0.01) in dogs fed HFD than in those consuming CD. The decrease in pH on d 16 was larger (*P* < 0.01) than other days (Figure 1B). Fecal scores increased (wetter feces) after diet transition. The increases in fecal scores were greater (*P* < 0.01) in dogs fed HFD vs. dogs fed CD, with the largest change from baseline appearing on d 20 (*P* < 0.01) (Figure 1C). Fecal DM decreased after diet transition (Figure 1D). Dogs consuming CD had larger decreases (*P* = 0.03) in fecal DM than dogs consuming HFD.

**Table 6:** Fecal characteristics, fecal bacteria counts, fecal IgA, fecal calprotectin, and serum lipopolysaccharide (LPS)-binding protein of yeast- or cellulose-supplemented dogs before diet transition (day 13 or 14)^a^.

	**Treatment**			
**Item**	**Cellulose**	**Yeast (375 mg/d)**	**Pooled SEM**	***P*-value**
Fecal characteristics				
Fecal pH	7.43	7.30	0.02	0.21
Fecal score^b^	2.15	2.08	0.03	0.69
Fecal dry matter (DM; %)	39.77	40.15	0.22	0.66
Fecal bacteria (CFU)^c^				
*E. coli* (log_10_/g DM)	6.76	6.71	0.06	0.82
*C. perfringens* (log_10_/g DM)	7.99	8.40	0.04	0.06
Fecal IgA (mg/g DM)	7.94	4.87	0.50	0.55
Fecal calprotectin (ng/g DM)	0.47	0.46	0.01	0.82
Serum LPS-binding protein (μmol/L)	186.58	200.74	6.52	0.44

a *Data are expressed as means ± pooled standard error of the mean, n = 24*.

b *Fecal scoring system: 1 = hard, dry pellets; small, hard mass; 2 = hard, formed, dry stool; remains firm and soft; 3 = soft, formed, and moist stool; 4 = soft, unformed stool; assumes shape of container; 5 = watery; liquid that can be poured*.

c *CFU, colony-forming units*.

### Fecal Bacteria, IgA, and Calprotectin

Fecal *E. coli*, IgA, and calprotectin were not affected by yeast treatment prior to diet transition (Table 6). Dogs supplemented with yeast product tended to have higher (*P* = 0.06) fecal *C. perfringens* counts than control dogs. After diet transition, fecal bacteria were changed by diet, but not yeast treatment. Fecal *E. coli* decreased in HFD-fed dogs but increased in CD-fed dogs (*P* < 0.05; Figure 1E). Fecal *C. perfringens* increased in dogs fed HFD, but decreased in dogs fed CD (*P* < 0.01; Figure 1F). The change in fecal IgA after diet transition was affected by treatment and diet. Yeast-supplemented dogs tended to have higher (*P* = 0.06) fecal IgA than the control dogs. In addition, fecal IgA increased (*P* < 0.01) in HFD-fed dogs, but decreased (*P* < 0.01) in CD-fed dogs (Figure 1G). Finally, fecal calprotectin increased after diet transition (Figure 1H). Dogs consuming CD tended to produce more (*P* = 0.06) calprotectin than dogs consuming HFD.

### Serum LPS-Binding Protein

Serum LPS-binding protein concentration was not affected by yeast treatment prior to diet transition (Table 6). After diet transition, serum LPS-binding protein increased, but the changes from baseline were not affected by treatment or diet (Figure 1I).

## Discussion

Diet transition occurs when pet owners purchase different products, which might result in GI discomfort. Yeast products may serve as a functional ingredient in dog foods and may protect animals from digestive upset due to its benefits on GI health. Here, we investigated the effects of yeast cell wall fraction on fecal characteristics and GI tract stability in adult dogs undergoing diet transition from a dry kibble diet to a high-fiber diet or canned diet. Our findings revealed that before diet transition, all indices except for fecal *C. perfringens* counts, were not altered by yeast supplementation. After diet transition, all measurements, except for fecal IgA concentration were not changed by yeast treatment. Fecal IgA concentrations tended to increase in yeast-supplemented dogs.

Diet transition led to significant changes to fecal characteristics and inflammatory biomarkers. Fecal pH decreased after dogs were fed HFD, which was expected. Lower fecal pH is due primarily to greater short-chain fatty acid concentrations as a result of fiber fermentation by microbiota in the hindgut (21, 22). Fecal scores increased and fecal DM decreased when dogs were fed both HFD and CD, indicating wetter and/or looser stools. Fecal calprotectin, a protein complex present in neutrophils, monocytes and reactive macrophages, has been reported to be increased in dogs with chronic diarrhea and chronic inflammatory enteropathies (23, 24). Here, we observed increased fecal calprotectin concentrations after diet transition, which reveals a slight elevation in GI inflammation. Finally, serum LPS-binding protein concentrations were elevated after diet transition. LPS binding-protein binds to endotoxins, including LPS from Gram-negative bacteria, with their concentration being increased after endotoxemia or bacteremia (25, 26). The increased serum LPS-binding protein concentrations in this study suggested greater GI permeability (leaky gut) after diet transition.

Fecal IgA was the only parameter that tended to be affected by yeast supplementation after diet transition. Elevated secretory IgA concentrations indicate enhanced mucosal immunity. Secretory IgA in the intestine plays a role in protecting mucosal surface against enteric antigens by inhibiting colonization and invasion of pathogens as well as food antigen uptake (27, 28). This response was in agreement with previous studies that reported elevated IgA concentrations in dogs consuming yeast products, indicating enhanced mucosal immunity. Middelbos et al. (4) noted that ileal IgA concentrations tended to respond quadratically (*P* = 0.09) to yeast cell wall supplementation in adult healthy dogs, with the highest concentration coming from the 0.25% supplementation level. Swanson et al. (11) supplemented 0.25% mannanoligosaccharides (MOS), the main component of the yeast cell walls, to adult healthy dogs and observed a tendency (*P* = 0.06) of increased ileal IgA concentrations. Increased IgA concentration may possibly be explained by two major components of the yeast cell walls, beta-glucans and MOS. Beta-glucans have been shown to be an immunostimulant that modulates innate and adaptive immune responses in various animal species, including dogs (29–31). As an immunostimulant, beta-glucans induce a cascade of immune responses by binding to dectin-1 receptors that are expressed on immune cells such as macrophages, monocytes, dendritic cells and neutrophils (32). The possible mechanisms by which MOS induce immune responses include the binding of mannose receptors that are expressed on antigen-presenting cells, including macrophage and dendritic cells (33). This response is supported by findings from previous studies in dogs, broilers, and pigs whereby oral administration of MOS led to enhanced immunity (34–37).

Supplementation of yeast products have been shown to decrease potential pathogenic bacteria such as *E. coli* in previous studies where dogs were supplemented with yeast cell wall, MOS or live *S. cerevisiae* (4, 5, 37). Decreased pathogenic bacteria are believed to be due to the ability of yeast cell wall to bind and inactivate bacteria. Mannose in yeast cell walls binds to type-1 fimbriae of pathogenic bacteria and thus prevent their adhesion and colonization to the host mucosa (38). We did not observe changes to fecal *E. coli* in this study, however, which is in line with findings from Swanson et al. (11) where dogs were supplemented with MOS. Although decreased fecal *C. perfringens* was noted in cats consuming yeast cell walls (7), lower fecal *C. perfringens* was not observed in dogs supplemented with MOS or live *S. cerevisiae* (5, 11, 37). Middelbos et al. (4) reported a cubic response of fecal *C. perfringens* counts to yeast cell wall supplementation in dogs, with greatest counts being observed at the 0.25% supplementation level. This is similar to our findings in dogs supplemented with 0.2% yeast cell wall fractions, who tended to have greater fecal *C. perfringens* counts compared to the control dogs before diet transition. Fecal *C. perfringens* counts in our study were 8.0–8.4 colony-forming units (CFU) log_10_/g DM, which is lower than those (9.5–10.0 CFU log_10_/g DM) reported by Middelbos et al. (4). The difference could be contributed by several factors, including diet composition, dog breed studied, and plating methods used to quantify microbes. The increased fecal *C. perfringens*, however, did not negatively affect measures of GI distress. *C. perfringens* are parts of commensal microbiota in dogs and can be cultured from more than 80% of healthy dogs and diarrheic dogs (39, 40). Therefore, greater fecal *C. perfringens* counts usually do not lead to diarrhea. Canine *C. perfringens*-associated diarrhea is more likely the result of intestinal microbiota disruption, which enables sporulation of commensal *C. perfringens* and production of enterotoxin (41). It also has been shown that *C. perfringens* enterotoxin is only detected in 5–15% of healthy dogs but in 34% of diarrheic dogs (39, 40). Fecal enterotoxin concentrations were not evaluated in the current study, but was unlikely because fecal scores were not affected by yeast cell walls.

The lack of significant effects on most intestinal health indices by yeast supplementation in this study could be due to the short treatment period used or the low supplement dosage tested. Grieshop et al. (37) observed decreased fecal *E. coli* counts in dogs supplemented with 1% MOS for 28 d. In dogs with enteropathogenic *E. coli*-induced diarrhea, supplementation of 2 g/kg BW MOS for 20 d resulted in faster recovery (42). Therefore, testing the yeast cell wall fractions with longer supplementation periods and/or higher dosages may be suggested in the future.

In conclusion, supplementation of 0.2% yeast cell wall fractions to dogs tended to increase fecal IgA concentrations, but did not affect fecal characteristics, fecal bacterial populations, or serum LPS-binding protein concentrations after an abrupt diet transition. Inclusion of yeast cell wall fractions in diets may modestly improve intestinal health for dogs undergoing diet transition.

## Data Availability Statement

The raw data supporting the conclusions of this article will be made available by the authors, without undue reservation.

## Ethics Statement

The animal study was reviewed and approved by the University of Illinois Institutional Animal Care and Use Committee.

## Author Contributions

KS and RR designed the experiment. C-YL and MM performed all laboratory analyses. C-YL and MC performed the animal trial and sample collection. C-YL conducted statistical analysis and wrote the manuscript. All authors have read and approved the manuscript.

## Conflict of Interest

RR was employed by Phileo by Lesaffre. The remaining authors declare that the research was conducted in the absence of any commercial or financial relationships that could be construed as a potential conflict of interest.
